# Query-Based Object Visual Tracking with Parallel Sequence Generation

**DOI:** 10.3390/s24154802

**Published:** 2024-07-24

**Authors:** Chang Liu, Bin Zhang, Chunjuan Bo, Dong Wang

**Affiliations:** 1 School of Information and Communication Engineering, Dalian University of Technology, Dalian 116024, China; njx2019@mail.dlut.edu.cn (C.L.); binzhangdlut@163.com (B.Z.); wdice@dlut.edu.cn (D.W.); 2 School of Information and Communication Engineering, Dalian Minzu University, Dalian 116600, China

**Keywords:** visual tracking, object tracking, transformer

## Abstract

Query decoders have been shown to achieve good performance in object detection. However, they suffer from insufficient object tracking performance. Sequence-to-sequence learning in this context has recently been explored, with the idea of describing a target as a sequence of discrete tokens. In this study, we experimentally determine that, with appropriate representation, a parallel approach for predicting a target coordinate sequence with a query decoder can achieve good performance and speed. We propose a concise query-based tracking framework for predicting a target coordinate sequence in a parallel manner, named QPSTrack. A set of queries are designed to be responsible for different coordinates of the tracked target. All the queries jointly represent a target rather than a traditional one-to-one matching pattern between the query and target. Moreover, we adopt an adaptive decoding scheme including a one-layer adaptive decoder and learnable adaptive inputs for the decoder. This decoding scheme assists the queries in decoding the template-guided search features better. Furthermore, we explore the use of the plain ViT-Base, ViT-Large, and lightweight hierarchical LeViT architectures as the encoder backbone, providing a family of three variants in total. All the trackers are found to obtain a good trade-off between speed and performance; for instance, our tracker QPSTrack-B256 with the ViT-Base encoder achieves a 69.1% AUC on the LaSOT benchmark at 104.8 FPS.

## 1. Introduction

**Figure 1:**
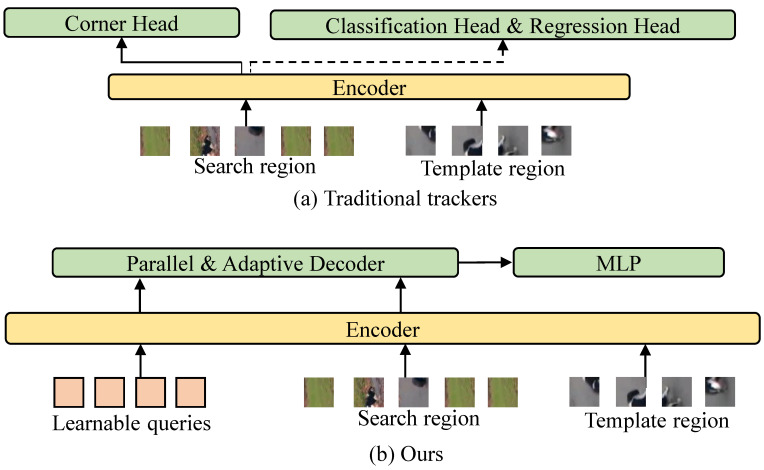
Comparison of proposed tracking framework with other representative trackers. Most trackers follow the tracking pipeline in (**a**). Our tracking framework, shown in (**b**), is a query-based tracking pipeline that adopts a parallel and adaptive decoder. The target is represented by a sequence of queries; each query is responsible for a coordinate, and all queries are predicted in parallel.

The visual object tracking (VOT) task aims to localize a target specified in the first frame in subsequent video frames. Benefiting from the transformer architecture, recent trackers have shown stronger feature representations and feature fusion modeling capabilities [1,2,3,4,5,6,7]. For the tracking head, as shown in Figure 1a, most trackers still adopt dense prediction and usually contain separate branches for target classification and regression [4,5,8,9,10] for later target bounding box selection. The corner head [2], which directly predicts the target bounding box through the spatial probability distribution of box corners, has recently achieved excellent performance. Although these tracking heads dominate, they require either elaborate design for a tracking task [2] or complicated joint localization of multiple prediction branches involving anchor design and inference post-processing [4,5,8,9,10].

DETR [11] adopts a query-based object detection method, which provides a new perspective to conduct object detection in the manner of direct set prediction. Queries represent the potential objects that are learnable, benefiting from attention mechanisms. A simple feed-forward network can predict the object coordinates and class labels based on queries after the decoding of image features. However, few works on single-object tracking have adopted query-based approaches for prediction. In STARK [2], the query token was only utilized for attention map computation and the score head. A longer time to convergence [7] and inferior performance compared with the conventional prediction heads [6] may be obstacles limiting the application of query-based heads in tracking tasks.

In this study, we propose a concise query-based tracking framework for the prediction of target coordinate sequences in a parallel manner, named QPSTrack. In contrast to the existing approaches, each query is no longer responsible for a target object but is instead responsible for a coordinate of the target object. As an object can be described using at least four coordinates, we provide a set of four queries to decode the template-enhanced search region features. As shown in Figure 1b, the overall framework adopts an encoder–decoder architecture. The encoder is a plain transformer that follows the one-stream pipeline, as in [5]. To make the decoding more adaptive to each template–search region pair, we introduce a one-layer adaptive decoder composed of a variant of the MLP-mixer [12] inspired by AdaMixer [13]. Meanwhile, we design adaptive inputs for the decoder by directing the learnable queries to the encoder backbone along with the template and search features. The queries updated by the encoder are utilized as the initial inputs of the decoder. As a whole, using an adaptive decoding scheme, four queries jointly represent a target and generate the coordinate sequences of the target in parallel. Furthermore, although there is an intuitive impression that the description of the location of the target is not limited by a strict order, the description format may have a strong influence. We also explored the differences in coordinate formats and adopt $\left[x_{min},y_{min},x_{max},y_{max}\right]$ as the best format.

Extensive experiments demonstrated that our tracker can achieve comparable performance with the state-of-the-art single-object tracking benchmarks without the use of any post-processing technique. Only plain cross-entropy loss is utilized to supervise training rather than the combination of $\ell_{1}$ loss and generalized IoU loss [14] specially designed for the bounding box regression task. Benefiting from the parallel sequence generation approach and light adaptive decoder, our tracker also achieves a good balance between tracking speed and performance. For instance, our QPSTrack tracker with a ViT-Base [15] backbone obtained a 69.1% AUC with 104.8 FPS on the LaSOT benchmark, being on par with OSTrack [5] in terms of accuracy and speed. In addition to the plain transformer backbone, we also explore our framework with a lightweight hierarchical backbone, LeViT [16]. Based on the light backbone, we propose a lightweight variant of the tracker, achieving a good balance of tracking performance and speed with simple multi-feature aggregation. We also explore the impact of the token format, representing the target information, on the sequence-to-sequence learning ability.

Our main contributions are summarized as follows:

- We propose a concise framework for query-based sequence generation tracking. A set of four queries are designed to represent the target’s localization, with each query being responsible for one of the target coordinates. This framework generates the target coordinate sequence with a query-based head operating in parallel.
- To make the decoding more adaptive to template-specific search region features from the perspectives of content and position, we adopt an adaptive decoding scheme including a one-layer adaptive decoder and learnable adaptive inputs for the decoder.
- We explore the ViT-Large, ViT-Base [17], and light LeViT [16] backbones to obtain a family of trackers. The experiments are conducted on popular benchmarks, and the results demonstrate that our framework can obtain comparable performance and achieve a good balance of performance and speed when compared with the state-of-the-art trackers.

## 2. Related Work

**Single-Object Tracking.** The existing trackers have undergone significant evolution, particularly benefiting from the transformer architecture. In terms of feature extraction, transformer architectures such as ViT [17] initialized with pre-trained weights—especially those pre-trained in a self-supervised manner with huge data, such as MAE [15]—can obtain stronger feature representations when compared with architectures such as ResNet [18]. In many trackers [2,4,7], the transformer architecture also replaces the previous complex fusion designs for better feature fusion. Further, benefiting from the flexible and inclusive inputs of the transformer, the one-stream tracking paradigm has been proposed [5,19]. Differing from the two-stream Siamese tracking paradigm, the one-stream approach combines the feature extraction and feature fusion of the template and search regions and is more effective. The template and search features can interact with each other in all the layers, leading to deep coupling.

Unlike the backbone and fusion module, the prediction head for target localization mainly adopts separate target classification and regression branches, the evolution of which is not significant. The target classification branch usually attempts to classify foreground–background candidate samples, selecting the index with respect to the maximum value in the response map. Then, the regression results with the corresponding index are selected to determine the bounding box coordinates [8]. In [5], the offsets were also predicted to compensate for the discretization error, and the weighted focal loss [20] was utilized for better classification. In [7], through the prediction of the IoU-aware classification score, the varifocal loss [21] was employed for classification. These methods are complicated in design, inevitably introducing some post-processing operations. As an exception, the corner head [2] can predict the target coordinates in an end-to-end manner through estimating the probability distribution of the target bounding box corners. However, it still requires a task-specific design involving stacked Conv-BN-ReLU layers. Although query-based detectors comprise a simple MLP prediction head for direct regression and achieve good performance, the pipeline is not widely utilized and may not work well in the object tracking field [2,6].

In this work, we propose a concise query-based tracking framework for predicting the target coordinate sequences in a parallel manner. We re-define the queries to represent the target coordinates and adopt an adaptive decoding scheme to assist the queries in decoding the target information for prediction. This framework can enable a query-based tracker to achieve comparable performance to the state-of-the-art trackers. A good trade-off between performance and speed is also achieved with our framework.

**Sequence-to-sequence Modeling.** Originating from the natural language processing field, sequence-to-sequence modeling has been applied in the computer vision field by some representative works. For instance, Pixe2Seq [22] models the object detection task as the generation of a sequence of discrete tokens representing object descriptions in an auto-regressive manner. In other words, given an image and preceding description tokens, the model is trained to predict the next description token. The box coordinates and class label are regraded as “language,” and the vocabulary size is set as the size of the defined discrete quantization space of continuous coordinates. In this modeling approach, the loss function and prediction head are more general among different tasks. Consequently, in this work, we adopt the idea of sequence-to-sequence modeling and likelihood maximization during training. However, compared with the typical sequence-to-sequence learning, the core idea of our work differs from two perspectives: (i) due to the intuition that the target coordinate description sequence should be unordered, we resort to parallel target sequence generation rather than the mode of predicting the next token one-by-one. Similar ideas have also emerged with regard to a query-based detector, in terms of utilizing the box coordinates as queries [23], which was shown to work well; and (ii) we adopt an adaptive decoder and the learnable adaptive queries are fed into the decoder as inputs. There is no need for a learnable vocabulary codebook for the mapping of discrete values.

## 3. Proposed Method

### 3.1. Overview

**Figure 2:**
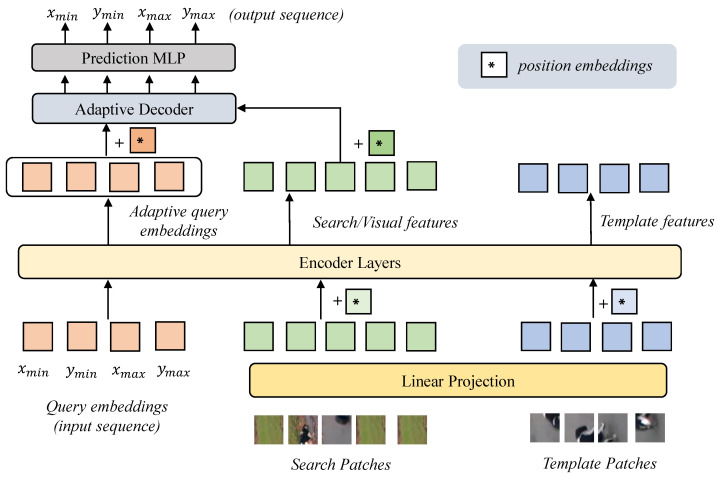
Overall architecture of the proposed tracker. The core component is the encoder–decoder transformer. Four additional queries, which represent four tokens of the target coordinates sequence, are fed into the encoder with the template–search pair. Then, the output queries are sent to the decoder as adaptive inputs. Finally, the adaptive decoder will decode the visual features to the queries and the prediction MLP will predict the target’s coordinate sequence.

The overall architecture of our proposed tracker is shown in Figure 2. The tracker employs an encoder–decoder architecture. The target bounding box in our tracker is represented as a sequence of $\left[x_{min},y_{min},x_{max},y_{max}\right]$, and tokens of the sequence are generated in a parallel, rather than sequential, order. Every token is represented by learnable query embeddings. The encoder follows the one-stream pipeline detailed in [5,19], with a plain vision transformer for feature extraction and feature fusion. Simultaneously, the query embeddings are concatenated with embedded features of search patches and template patches. The concatenated features are fed into encoder to provide the learnable adaptive query embeddings, in a manner dependent on the template–search pair as the decoder’s initial query inputs. The query-based decoder is an adaptive decoding module with a variant of the MLP-mixer architecture [12], as in AdaMixer [13]. The decoder receives the adaptive query embeddings as the input token sequence and adopts dynamic weights generated seperately for every input token, enabling feature mixing. The decoded query embeddings are sent to a multi-layer perceptron (MLP) to generate the final target coordinates sequence. Further details are provided in the following.

### 3.2. Network Architecture

**Figure 3:**
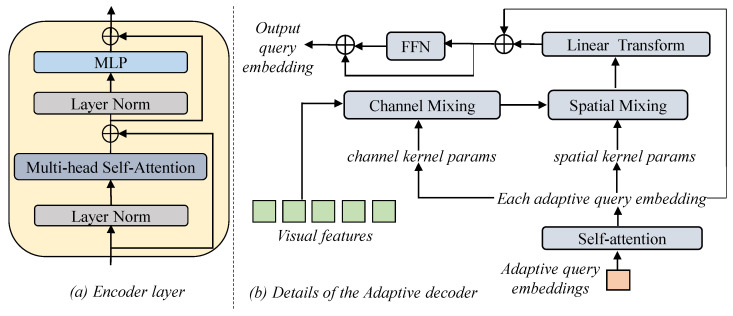
(**a**) Details of each encoder layer. (**b**) Details of the adaptive decoder. The generated parameters are dependent on each adaptive query for spatial and channel mixing.

**Encoder.** The encoder is the plain vision transformer architecture, disposing of the class embedding. The template regions $I_{z}\in R^{3*H_{z}*W_{z}}$ and search regions $I_{x}\in R^{3*H_{x}*W_{x}}$ are first divided into a sequence of patches. Then, all the patches will be embedded into d-dimensional patch embeddings, obtaining $H_{x}\in R^{m*d}$ and $H_{z}\in R^{n*d}$ separately, using a shared trainable linear projection layer. Here, m,n are the number of patches for the search and template regions, respectively. To represent the four tokens of target coordinates sequence $\left[x_{min},y_{min},x_{max},y_{max}\right]$, we additionally introduce learnable query embeddings $H_{query}\in R^{4*d}$ to generate adaptive inputs for the query-based decoder. All the features of patches and learnable query embeddings $H_{query}$ are concatenated as $H_{xz}=\left[H_{query},H_{x},H_{z}\right]$. Then, the features $H_{xz}$ and corresponding learnable position embeddings will be fed into the transformer encoder layers $E_{l}$ for feature extraction and feature fusion:

(1) $$\begin{array}{cc} H_{xz}^{l+1} & =E_{l}\left(H_{xz}^{l};W_{l}\right)\\ H_{query}^{l+1},H_{x}^{l+1}, & H_{z}^{l+1}]=H_{xz}^{l+1},l=0,1,2\ldots ,L-1 \end{array}$$

where $W_{l}$ represents the weights of the $l^{\mathrm{th}}$ layer and *L* is the total number of encoder layers. The encoder layer mainly contains layer normalization, a self-attention module, and a multi-layer perceptron (MLP), as detailed in Figure 3a. The final layer’s output, $H_{query}^{L}$, will be sent to the decoder as the conditional initial query input rather than the query embeddings (which were initialized as zeros in DETR) [11]. This provides a frame-specific initialization of the decoder’s input, thus enhancing the decoding ability of different search features modulated by different target templates.

**Adaptive decoder.** Our decoder is designed in an adaptive manner in order to decode the template-guided search features, also known as visual features. As shown in Figure 2 and Figure 3b, the initial input sequence of decoder is trained to be adaptive to each template–search pair, and the decoding parameters are dependent on each token in the input sequence. Overall, the decoding process can better deal with the variations in different target-specific search features, and can assist in capturing the target to be tracked in training more quickly.

As mentioned in part of the encoder, we represent the target bounding box as a sequence with the format $\left[x_{min},y_{min},x_{max},y_{max}\right]$, with each token focusing on predicting the key information of different coordinates. Each token is represented by one query embedding, and a one-layer self-attention module is first introduced between these queries. Then, as in [13], a variant of MLP-mixer [12] is introduced for decoding. Dynamic mixing weights dependent on each token are generated for adaptive location and content decoding. The channel kernel parameters $K_{c}\in R^{C*C}$ and spatial kernel parameters $K_{s}\in R^{C_{in}*C_{out}}$ are generated as the sum of each query embedding $H_{query}^{i}\in R^{1*C},i\in \left[0,1,2,3\right]$ and corresponding learnable token position embedding $P_{query}^{i}\in R^{1*C},i\in \left[0,1,2,3\right]$, enabling channel and spatial mixing:

(2) $$\begin{array}{c} H_{query}=SelfAttn\left(H_{query}+P_{query}\right)\\ K_{c^{i}}{=Linear}_{c}\left(H_{query}^{i}\right)\\ K_{s^{i}}{=Linear}_{s}\left(H_{query}^{i}\right) \end{array}$$

Then, under the guidance of each query, the visual features $H_{x}\in R^{m*d}$ with the learnable position embeddings $P_{x}\in R^{m*d}$ are adaptively decoded based on the corresponding focus:

(3) $$\begin{array}{cc} O_{mixed_{c}}=ReLU(LayerNorm & \left(H_{x}+P_{x}\right)K_{c^{i}})\\ O_{mixed_{s}}=ReLU(LayerNorm & \left(O_{mixed_{c}}^{T}K_{s^{i}}\right)\\ O_{add}=Linear(O_{mixed_{s}} & )+H_{query}^{i}\\ H_{query}^{i^{\prime }}=FFN(O_{add} & )+O_{add} \end{array}$$

where *C* is the channel number of visual features and query embeddings, $C_{in}$ is the number of visual features, $C_{out}$ is the number of spatial mixing out patterns, $O_{mixed_{c}}$ is the output of channel mixing, $O_{mixed_{s}}$ is the output of spatial mixing, $O_{add}\in R^{1*C}$ is the residual output of query embeddings, FFN represents a feed-forward network composed of a two-layer perceptron, and $H_{query}^{i^{\prime }},i\in \left[0,1,\ldots ,3\right]$ represents the final updated query embeddings after decoding. Then, as in the default query-based manner, a multi-layer perceptron (MLP) prediction head is applied to the decoded query embeddings $H_{query}^{\prime }$ for prediction of the final target coordinate distribution. Every layer of the multi-layer perceptron consists of a linear projection layer and a ReLU activation function, except for the final layer.

**Multi-scale features.** Lightweight backbones often adopt hierarchical structures, resulting in a lower-resolution feature map in the last layer and making it difficult to follow tracking prediction heads. For better performance, we apply transpose convolutional layers to up-sample the resolution of all features and align them with the first stage, following which all features are summed in an element-wise manner, as follows:

(4) $$\begin{array}{cc} H_{x}^{L-i}=Linear & \left(Upsample\left(H_{x}^{L-i}\right)\right)\\ H_{x}^{final}= & \sum_{i=0}^{k}\left(H_{x}^{L-i}\right) \end{array}$$

where *k* is the stage number of the hierarchical backbone and *L* is the total number of the scale features.

### 3.3. Training

In this work, we adopted the cross-entropy loss function, as used in many sequence-to-sequence learning methods [22], for training of the overall network. All the continuous coordinate regression values are converted to discretized integers uniformly distributed within the interval [1,nbins]. Every target ground-truth can be represented by four token coordinates $\left[x_{min},y_{min},x_{max},y_{max}\right]$. For the network, we attempt to maximize the log-likelihood of all target tokens represented by the decoded query embeddings.

## 4. Experiments

### 4.1. Implementation Details

**Model Details.** We provide three variants of the proposed tracker, **QPSTrack-B256**, **QPSTrack-L256**, and **QPSTrack-Light**. In contrast to the first two variants, **QPSTrack-Light** is a lightweight variant. For all variants, the template region was resized to 128×128 pixels, cropped to 2^2^ times the target area; the search region was resized to 256×256 pixels, cropped to 4^2^ times the target area.

**QPSTrack-B256** adopts the ViT-Base [17] model as the encoder backbone, while **QPSTrack-L256** adopts the ViT-Large [17] model as the encoder backbone. The final prediction head is a simple three-layer perceptron, used to decode the output query embeddings to final target coordinates. The hidden dimension of the three-layer perceptron head is consistent with the input dimension of the head; namely, 256. The output dimension of the three-layer perceptron head is nbins, which was set to 1000. In addition, the hyperparameter $C_{out}$ was set to 128 and *C* was set to 256.

**Training Details.** Our tracker was implemented using PyTorch 1.7.0. The MAE [15] pre-trained parameters were utilized to initialize the encoder backbones for both the ViT-Base [17] and ViT-Large [17] architectures. Aligned with the normal training settings in the single-object tracking works [2,5], the training data included the training splits of COCO-2017 [24], LaSOT [25], GOT-10k [26], and TrackingNet [27], and brightness jitter and horizontal flip were adopted for data augmentation. The whole training process took a total of 300 epochs, with each epoch including $6\times 10^{4}$ sets of image pairs. The AdamW for the encoder and 2$\times \;10^{-5}$ for the remaining parts. The learning rate decreased by a factor of 0.1 for all parameters at the 240th epoch. Training was conducted on two NVIDIA A100 GPUs, where each GPU held a batch size of 64.

For the lightweight backbone, we adopted hierarchical LeViT [16] in order to explore the effectiveness of our framework. For simplicity, we only used the training splits of COCO-2017 [24], LaSOT [25], and GOT-10k [26] as training data. The training is conducted on two NVIDIA 3090 GPUs for 500 epochs, with each GPU having a batch size of 64. The optimizer was AdamW [28]. The learning rate was set to 5 $\times \;10^{-4}$ for the encoder, and 5 $\times \;10^{-5}$ for the remaining parts. Due to the incompatible backbone architecture for additional learnable query embeddings as encoder inputs, adaptive queries were not performed with the lightweight variant.

**Inference.** The inference speed was calculated on an NVIDIA RTX 2080Ti GPU with Intel Core i9-9900KF CPU @ 3.60 Hz × 16. The whole inference was conducted in an end-to-end fashion, without any post-processing operations such as Hanning windowing or template updating.

### 4.2. Benchmark Evaluation

To verify the effectiveness of our framework, we compared our proposed trackers **QPSTrack-B256** and **QPSTrack-L256** on six popular single-object tracking benchmarks with the state-of-the-art (SOTA) trackers. The detailed results and analysis are discussed in the following.

**Figure 4:**
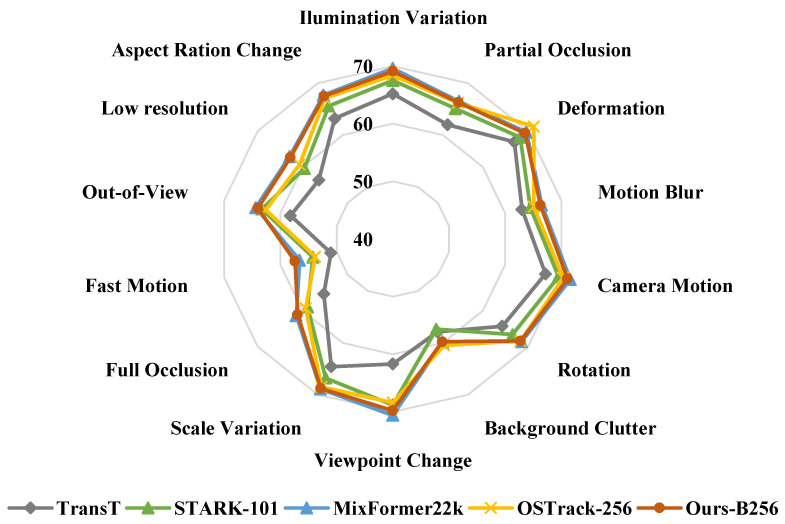
AUCs for different attributes on LaSOT [25]. Our tracker can be seen to be competitive in multiple attributes, especially with respect to the ‘Fast Motion’ attribute.

**LaSOT.** The LaSOT [25] is a popular and large benchmark with 280 videos for the testing split. The overall results are reported in the first column of Table 1. OSTrack [5] predicts the center classification map, offset, and target size jointly [5], while SimTrack [19] adopts the prediction head as in STARK [2] with foveal window strategy. Without any post-processing, such as multiple templates or updating, the QPSTrack-B256 tracker obtained an AUC of 69.1%, achieving comparable performance to OSTrack-256 [5] and SimTrack-B [19], which also adopt the ViT-Base backbone and have similar input resolutions. Moreover, the QPSTrack-B256 tracker ran at a faster speed of 104.8 FPS. The inference speed was on par with OSTrack-256 [5], with early candidate elimination to enhance its speed, and more than twice that (about 40 FPS) of SimTrack [19]. The QPSTrack-L256 tracker outperformed SimTrack-L [19] by 0.4%, with an AUC of 70.6% and running at a speed of 31.6 FPS. We also report the AUC on different attributes for the QPSTrack-B256 tracker in Figure 4. Our tracker demonstrated good competitiveness in all the various attributes when compared with other trackers.

**LaSOT_*ext.*_** As an extension of LaSOT [25], ${\mathrm{LaSOT}}_{ext}$ [29] provides an additional 150 videos in 15 other classes that do not intersect with the categories of LaSOT’s training set. We used the python toolkit provided by OSTrack [5], rather than the MATLAB toolkit, for evaluation. The comparative results are reported in the second column of Table 1. The QPSTrack-B256 tracker achieved 47.0% AUC on the ${\mathrm{LaSOT}}_{ext}$ [29] benchmark, with only a slight disadvantage of 0.4% compared to OSTrack-256. The QPSTrack-L256 tracker also achieved good performance, with an AUC of 49.1%.

**TrackingNet.** A total of 511 videos are provided in the TrackingNet [27] benchmark. As shown in the third column of Table 1, QPSTrack-B256 tracker lagged 1.7% behind OSTrack-256 [5], while the QPSTrack-L256 tracker surpassed SimTrack-L [19] by 0.1% in AUC and was on par with the latter in the normalized precision metric.

**GOT-10k.** The GOT-10k [26] benchmark contains 180 videos for testing. We followed the principle of training the model using only the training data of GOT-10k rather than all the training data. Only 100 epochs were carried out, in alignment with the normal settings in [5], and the learning rate was decreased at the 80th epoch. As reported in the rightmost column of Table 1, the QPSTrack-B256 tracker achieved an AO metric of 68.0%, while QPSTrack-L256 achieved an AO of 71.2% and surpassed SimTrack-L [19] by 1.4% in terms of AUC. With the larger encoder, our tracker’s performance showed an increase of approximately 3.2%, outperforming the otherwise leading model SimTrack [19].

**UAV123 and NFS.** UAV123 [43] contains 123 videos captured by drones, while the NFS [44] benchmark contains 100 videos. The results are shown in Table 2, from which it can be seen that both of our tracker variants achieved comparable performance with the state-of-the-art trackers, indicating the effectiveness and generalization ability of our framework.

**Speed and Number of Parameters.** As detailed in Table 3, our tracker with the ViT-Base backbone and 256 × 256 resolution ran at around 104.8 fps, which is close to the speed of OSTrack [5], which uses the candidate elimination strategy to improve its speed. Compared with other representative trackers such as SimTrack-B [19] and MixFormer [6], our tracker also presented a significant speed advantage. After replacing the backbone with the ViT-Large [17] architecture, the speed reached 31.6 FPS, which is still in real time. The overall results show that our tracker can achieve a good balance of tracking speed and performance due to the parallel sequence generation approach and concise decoder architecture designs.

### 4.3. Ablation Studies

All the ablation studies were conducted with the QPSTrack-B256 variant tracker on the LaSOT [25] benchmark.

**Figure 5:**
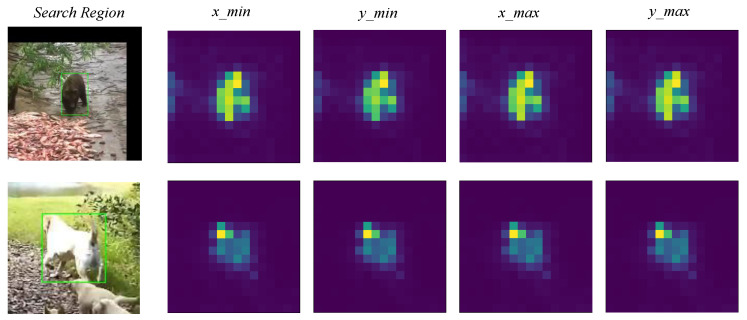
Visualization of the attention weights of the search region corresponding to each query token. Target in the search region is in green bounding box.

**Component-wise Analysis.** We evaluated the effectiveness of the different components in our tracker, and the results are shown in Table 4. When only the adaptive decoder was utilized—in other words, the input of decoder was the default zero-initialized decoder as in DETR [11], rather than the adaptive query inputs—the performance dropped by 0.6% in the AUC metric, as shown in the second line. When the adaptive inputs were fed into final prediction MLP directly, with the adaptive decoder removed, the encoder–decoder architecture degraded into an encoder-only architecture. For this model, the performance declined by 1.7% on the LaSOT benchmark, as shown in the first line of the table, demonstrating the importance of the adaptive decoder. Meanwhile, the results also indicate that the adaptive query embeddings can already enable relatively strong representation ability while attending to template–search features in the encoder due to the powerful attention mechanism. We visualized the attention weights of the search region corresponding to each query token of the ViT-Base [17] architecture’s last layer, as shown in Figure 5, from which it can be seen that the adaptive queries have already obtained discriminative attention on the target.

**Token Format.** For the target coordinate sequence, there are two common token formats for representation: $\left[x_{min},y_{min},w,h\right]$ and $\left[x_{min},y_{min},x_{max},y_{max}\right]$. We also tested the token representation format $\left[x_{min},y_{min},w,h\right]$ for comparison. As shown in Table 5, the performance dropped sharply (by 6.6%) on the LaSOT [25] benchmark. We conjecture that the parallel sequence generation approach prevents serious interdependence of the tokens of sequence, such that every token of $\left[x_{min},y_{min},x_{max},y_{max}\right]$ can focus on the content with absolute location information. Meanwhile, for [w,h], only under the conditions provided by $\left[x_{min},y_{min}\right]$ can the [w,h] tokens and $\left[x_{min},y_{min}\right]$ tokens jointly determine a target box. Thus, the tokens are difficult to decouple from causal relationships, resulting in bad localization performance.

When the tracking confidence score is required, we add an extra token ‘IoU’ to predict the IoU value for tracking quality prediction. This token can be appended to the target coordinate sequence directly. In other words, the target can be represented in the token format $\left[x_{min},y_{min},x_{max},y_{max},IoU\right]$ to additionally predict the regression quality prediction additionally. The ‘IoU’ token can be supervised with the mean-squared error loss $\ell_{mse}$. $L=\lambda_{mse}\ell_{mse}+\lambda_{ce}L_{ce}$. Both weights of loss functions are set to 1, and we report the results of this joint training in Table 5. On the LaSOT benchmark, adding the IoU token led to a 0.7% decrease in the AUC, demonstrating that joint training has a negative effect on the performance.

**Loss functions and Decoder.** Common tracking algorithms adopt task-specific loss functions, including the combination of $\ell_{1}$ loss and generalized IoU loss [14], for localization supervision. Inspired by sequence-to-sequence learning, the cross-entropy loss, which is a more general loss function among sequence tasks, can also be utilized for trackers. To evaluate the impact of the task-specific loss and cross-entropy loss on performance, we replaced the cross-entropy loss in our tracker with the combined loss. The output dimension of the final prediction MLP was set as 1 for direct normalized value prediction rather than nbins. As shown in Table 5, the combined loss function led to a 1.2% decrease in AUC and a 1.0% decrease in precision, while the normalized precision metric was not affected. Although the parallel sequence generation can be compatible with different loss function designs, the results show that the tracking-specific loss may not necessarily have performance advantages over the simple cross-entropy loss in our framework.

**Figure 6:**
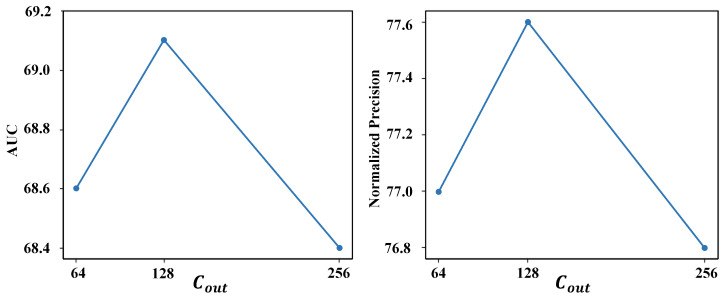
Impact of $C_{out}$ in spatial mixing on LaSOT [25]. AUC and normalized precision are reported separately.

Meanwhile, we replaced the adaptive decoder with a one-layer plain decoder [11] for comparison. The AUC performance dropped by 0.7% on the LaSOT benchmark. We also explored the impact of the parameter $C_{out}$ in the spatial mixing operation on the performance. We tested values of 64, 128, and 256 separately. As shown in Figure 6, increasing the value of $C_{out}$ does not necessarily provide performance benefits. As a result, we set $C_{out}$ to 128 for the best performance.

### 4.4. Lightweight Backbone

**Figure 7:**
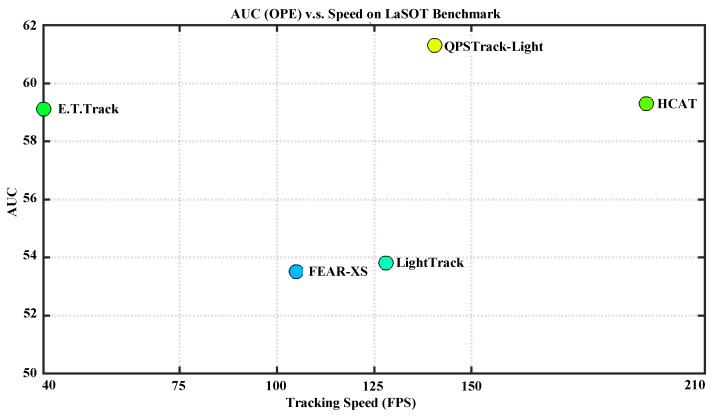
Speed and performance comparison on LaSOT [25]. Our QPSTrack-Light achieved 140.6 fps while exceeding HCAT [45] by 2.0% AUC.

**Figure 8:**
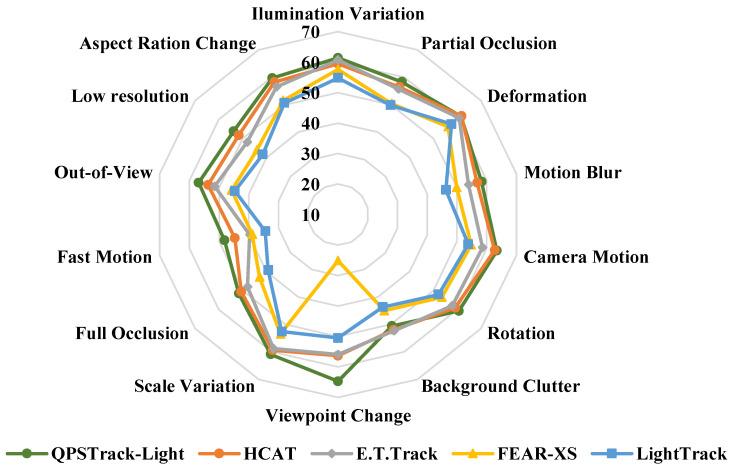
AUCs of different attributes on LaSOT [25] compared with other lightweight trackers. Our tracker showed significant advantages in the ‘Viewpoint Change’, ‘Out-of-View,’ and ‘Fast Motion’ attributes.

We also explored the use of a lightweight encoder backbone to verify the effectiveness of our tracking framework. Using the lightweight hierarchical backbone LeViT [16], the performance is reported in Table 6. Experiments were conducted on the LaSOT [25] benchmark. The details of the lightweight tracker are shown in the third row of Table 3. The reported results show that our tracking framework is also compatible with a lightweight hierarchical backbone, indicating good performance in comparison with all the lightweight trackers. As shown in Figure 7, our QPSTrack-Light exceeded other lightweight trackers in terms of performance while also presenting a good advantage in speed over most of its competitors. We also report the AUC on different attributes of the QPSTrack-Light tracker, in comparison with other lightweight trackers, in Figure 8. Our tracker presented significant advantages with respect to the ‘Viewpoint Change’, ‘Out-of-View,’ and ‘Fast Motion’ attributes.

### 4.5. Limitations

As shown in Table 1 and Table 2, the obtained precision metrics indicated some disadvantages, when compared to other trackers, and the AUC performance on some data sets was still not very ideal, such as TrackingNet [27]. This implies that further steps are needed to explore means for improving the obtained performance. Additionally, while the proposed tracker provides a concise tracking framework for generating the target coordinates sequence in parallel, there is insufficient utilization of temporal information, which can be explored further in future works.

## 5. Conclusions

In this study, a concise tracking framework for query-based sequence generation was proposed. The target coordinates are represented with queries, where each query is responsible for predicting one coordinate. All the coordinates $\left[x_{min},y_{min},x_{max},y_{max}\right]$ are predicted in parallel using the query-based head. For better decoding of the search features to predict the target localization, an adaptive decoder is adopted. The decoder is fed with the learnable queries as adaptive inputs, and the learnable queries are obtained by attending to the encoder backbone, along with the template and search region features. The decoding scheme provides more adaptability to different template–search pairs in order to deal with more diverse target variations. We construct a family of trackers with different backbones, including the light backbone LeViT. The experimental results on multiple benchmarks indicate that our trackers achieve comparable performance to the state-of-the-art trackers while maintaining high speed. Meanwhile, the analysis shows that the target coordinates represented in $\left[x_{min},y_{min},x_{max},y_{max}\right]$ format enable the trackers carrying out unordered parallel prediction in sequence-to-sequence learning to achieve good performance. 

## Figures and Tables

**Table 1:** Comparison with state-of-the-art models on the LaSOT [25], LaSOT_*ext*_ [29], GOT-10k [26], and TrackingNet [27] benchmarks.

Tracker	LaSOT			${\mathrm{LaSOT}}_{\mathrm{ext}}$			TrackingNet			GOT-10k		
	AUC	$P_{\mathrm{norm}}$	p	AUC	$P_{\mathrm{norm}}$	p	AUC	$P_{\mathrm{norm}}$	p	AO	SR_0.5_	SR_0.75_
QPSTrack-L256	70.9	80.0	76.9	49.1	58.4	54.5	83.5	87.4	82.4	71.2	79.5	68.9
SimTrack-L [19]	70.5	79.7	-	-	-	-	83.4	87.4	-	69.8	78.8	66.0
QPSTrack-B256	69.1	77.6	73.9	47.0	55.5	51.4	81.6	85.4	79.3	68.0	76.0	63.7
SwinTrack-B [7]	69.6	78.6	74.1	47.6	58.2	54.1	82.5	87.0	80.4	69.4	78.0	64.3
SimTrack-B [19]	69.3	78.5	-	-	-	-	82.3	86.5	-	68.6	78.9	62.4
MixFormer-22k [6]	69.2	78.7	74.7	-	-	-	83.1	88.1	81.6	70.7	80.0	67.8
OSTrack-256 [5]	69.1	78.7	75.2	47.4	57.3	53.3	83.1	87.8	82.0	71.0	80.4	68.2
AiATrack [30]	69.0	79.4	73.8	47.7	55.6	55.4	82.7	87.8	80.4	69.6	63.2	80.0
ToMP-101 [31]	68.5	-	-	45.9	-	-	81.5	86.4	78.9	-	-	-
GTELT [32]	67.7	-	73.2	45.0	54.2	52.4	82.5	86.7	81.6		-	
KeepTrack [33]	67.1	77.2	70.2	48.2	58.1	56.4	-	-	-	-	-	-
STARK-101 [2]	67.1	77.0	-	-	-	-	82.0	86.9	-	68.8	78.1	64.1
TransT [4]	64.9	73.8	69.0	-	-	-	81.4	86.7	80.3	67.1	76.8	60.9
SiamR-CNN [34]	64.8	72.2	-	-	-	-	81.2	85.4	80.0	64.9	72.8	59.7
UTT [35]	64.6	-	67.2	-	-	-	79.7	-	77.0	67.2	76.3	60.5
TrDiMP [1]	63.9	-	61.4	-	-	-	78.4	83.3	73.1	67.1	77.7	58.3
DMTrack [36]	58.4	-	59.7	-	-	-						
LTMU [37]	57.2	-	57.2	41.4	49.9	47.3	-	-	-	-	-	-
GlobalTrack [38]	51.7	-	52.8	35.6	43.6	41.1	70.4	75.4	65.6	-	-	-
SiamPRN++ [8]	49.6	56.9	49.1	34.0	41.6	39.6	73.3	80.0	69.4	51.7	61.6	32.5
SPLT [39]	42.6	-	39.6	27.2	33.9	29.7						
ECO [40]	32.4	33.8	30.1	22.0	25.2	24.0	55.4	61.8	49.2	31.6	30.9	11.1
MDNet [41]	39.7	46.0	37.3	27.9	34.9	31.8	60.6	70.5	56.5	29.9	30.3	9.9
SiamFC [42]	33.6	42.0	33.9	23.0	31.1	26.9	57.1	66.3	53.3	34.8	35.3	9.8

**Table 2:** Comparison with the state-of-the-art models on the UAV123 and NFS benchmarks. The AUC and precision scores are reported.

Tracker	UAV123		NFS	
	AUC	P	AUC	P
QPSTrack-L256	68.7	89.8	64.6	80.1
QPSTrack-B256	67.5	87.8	63.1	76.8
MixFormer-22k [6]	70.4	91.8	-	-
SimTrack-B [19]	69.8	89.6	-	-
KeepTrack [33]	69.7	-	66.4	-
OSTrack-256 [5]	68.3	-	64.7	-
STARK-101 [2]	68.2	-	66.2	-
TransT [4]	68.1	87.6	65.3	78.8
TrDiMP [1]	66.4	86.9	66.2	79.1
SiamR-CNN [34]	64.9	83.4	63.9	-
SiamPRN++ [8]	59.3	78.2	57.1	69.3

**Table 3:** Speed, MACs, and Params of different variants of our proposed trackers and other representative trackers. ‘^†^’ denotes the speed reported in the original papers.

Tracker	MACs	Params	Speed (FPS)	
	(G)	(M)	GPU	CPU
QPSTrack-L256	98.4	337.8	31.6	-
QPSTrack-B256	27.9	120.3	104.8	-
QPSTrack-Light	6.64	83.8	140.6	24.6
SwinTrack-B [7]	-	91	${52\;}^{†}$	-
SimTrack-B [19]	22.3	88.1	$40.{0\;}^{†}$	-
OSTrack-256 [5]	21.5	92.1	$105.{4\;}^{†}$	-
STARK [2]	12.8	28.2	$41.{8\;}^{†}$	-
TransT [4]	19.2	28.4	${50\;}^{†}$	-

**Table 4:** Component-wise ablation studies on LaSOT [25].

*Adaptive Inputs*	*Adaptive Decoder*	LaSOT		
		AUC	$P_{\mathrm{norm}}$	P
✓		67.4	75.9	71.4
	✓	68.5	77.0	73.0
✓	✓	69.1	77.6	73.9

**Table 5:** Ablation studies on different loss functions, token representation format, and decoder on the LaSOT benchmark [25].

	Settings	LaSOT		
		AUC	Normalized Precision	Precision
*Loss Function*	cross-entropy loss	69.1	77.6	73.9
	task-specific loss	$67.9_{-1.2\%}$	$77.6_{-0.0\%}$	$72.9_{-1.0\%}$
	$\left[x_{min},y_{min},x_{max},y_{max}\right]$	69.1	77.6	73.9
*Token Format*	$\left[x_{min},y_{min},w,h\right]$	$62.5_{-6.6\%}$	$70.8_{-6.8\%}$	$66.5_{-7.4\%}$
	$\left[x_{min},y_{min},x_{max},y_{max},IoU\right]$	$68.4_{-0.7\%}$	$76.8_{-0.8\%}$	$72.9_{-1.0\%}$
*Decoder*	Adaptive decoder	69.1	77.6	73.9
	Plain decoder	$68.4_{-0.7\%}$	$77.0_{-0.6\%}$	$72.8_{-1.1\%}$

**Table 6:** Comparison with other lightweight trackers on the LaSOT [25] benchmark.

Tracker	LaSOT		
	AUC	$P_{\mathrm{Norm}}$	P
**QPSTrack-Light**	61.3	67.5	62.1
HCAT [45]	59.3	68.7	61.0
E.T.Track [46]	59.1	-	-
LightTrack [47]	53.8	-	53.7
FEAR-XS [48]	53.5	-	54.5

## Data Availability

Data are contained within the article.
